# HYFF-CB: Hybrid Feature Fusion Visual Model for Cargo Boxes

**DOI:** 10.3390/s25061865

**Published:** 2025-03-17

**Authors:** Juedong Li, Kaifan Yang, Cheng Qiu, Lubin Wang, Yujia Cai, Hailan Wei, Qiang Yu, Peng Huang

**Affiliations:** 1 College of Information Engineering, Guilin Institute of Information Technology, Guilin 541004, China; liyudong@guit.edu.cn (J.L.); yangkaifan@guit.edu.cn (K.Y.); 2120211049@glut.edu.cn (L.W.); caiyujia@guit.edu.cn (Y.C.); 2 Guangxi Zhuang Autonomous Region Institute of Metrology and Test, Nanning 530200, China; 3 National Space Science Center, Chinese Academy of Sciences, Beijing 100190, China; 4 College of Mechanical and Control Engineering, Guilin University of Technology, Guilin 541006, China; 2016014@glut.edu.cn

**Keywords:** automatic loading and unloading, box detection, machine learning, synergistic weighted loss

## Abstract

In automatic loading and unloading systems, it is crucial to accurately detect the locations of boxes inside trucks in real time. However, the existing methods for box detection have multiple shortcomings, and can hardly meet the strict requirements of actual production. When the truck environment is complex, the currently common models based on convolutional neural networks show certain limitations in the practical application of box detection. For example, these models fail to effectively handle the size inconsistency and occlusion of boxes, resulting in a decrease in detection accuracy. These problems seriously restrict the performance and reliability of automatic loading and unloading systems, making it impossible to achieve ideal detection accuracy, speed, and adaptability. Therefore, there is an urgent need for a new and more effective box detection method. To this end, this paper proposes a new model, HYFF-CB, which incorporates key technologies such as a location attention mechanism, a fusion-enhanced pyramid structure, and a synergistic weighted loss system. After real-time images of a truck were obtained by an industrial camera, the HYFF-CB model was used to detect the boxes in the truck, having the capability to accurately detect the stacking locations and quantity of the boxes. After rigorous testing, the HYFF-CB model was compared with other existing models. The results show that the HYFF-CB model has apparent advantages in detection rate. With its detection performance and effect fully meeting the actual application requirements of automatic loading and unloading systems, the HYFF-CB model can excellently adapt to various complex and changing scenarios for the application of automatic loading and unloading.

## 1. Introduction

In the context of the digital era, the development of Internet technology has significantly promoted the vigorous development of e-commerce, which, in turn, has given rise to the need for the transformation of logistics management into intelligent and efficient logistics. All aspects of the logistics industry, including the receipt, storage, and distribution of goods, require accurate and real-time management based on cost control [1,2]. As key nodes in the logistics process, loading and unloading have a direct impact on the goods turnover rate and warehousing efficiency [3,4,5]. Traditional loading and unloading processes, relying on manual operations, have limitations in efficiency and cost. With the growth of business volume, manual operations are characterized by high cost and increased error rates, affecting the overall logistics efficiency. Therefore, automatic loading and unloading systems have emerged. These systems can automatically complete loading and unloading tasks, including automatically identifying goods, calculating optimal loading solutions, and using automated equipment such as robotic arms for loading and unloading. However, existing automatic systems still face challenges when handling non-standardized goods [6,7].

In the field of modern logistics, the application of image processing and machine vision technologies is becoming increasingly widespread [8,9,10,11]. These technologies optimize the warehouse management process by providing accurate identification and classification of goods. The machine vision system, simulating human vision, captures images with cameras and uses algorithms for image analysis and processing to achieve automatic identification, localization, or classification of goods. The application of this technology has significantly improved the automation level of loading and unloading, reduced dependence on manual operations, and improved operating efficiency and accuracy [12,13,14]. Compared with traditional manual loading and unloading, automatic loading and unloading detection algorithms based on machine vision can achieve all-weather operations and reduce labor intensity and human errors, thereby ensuring the continuity and stability of logistics operations. However, existing cargo box detection methods still have certain limitations in practical applications. For example, when facing situations such as inconsistent cargo box sizes, occlusion, light changes, and complex backgrounds, the detection performance drops significantly. This severely restricts the efficiency and reliability of the automatic loading and unloading system [15,16,17].

In recent years, the successful applications of deep learning algorithms (such as SSD, YOLO, etc.) in the fields of object detection and image recognition have provided powerful technical support for logistics automation systems. By combining convolutional neural networks (CNNs) and feature pyramid networks (FPNs), these algorithms can achieve accurate detection of multi-scale objects, thereby improving the efficiency and reliability of automated loading and unloading systems. In the application of object detection, the YOLO (You Only Look Once) series of algorithms has attracted much attention [18,19,20,21]. The YOLOv8 algorithm, by introducing an improved feature pyramid structure and a dynamic anchor assignment mechanism, has not only improved the detection accuracy of the model, but also significantly enhanced its adaptability to multi-scale objects and complex environments [22,23]. However, when facing the special requirements of cargo box detection in an automatic loading and unloading system, the YOLOv8 algorithm has certain limitations. For example, in terms of the accurate positioning of cargo boxes and the detection of their quantity, YOLOv8 still has room for improvement when dealing with complex occlusions and diverse size distributions.

In order to fill the many gaps existing in the above-mentioned systems or algorithms, solve the problems of cargo box positioning and grasping during loading and unloading, improve logistics efficiency, and reduce warehousing costs, this paper proposes a new cargo box detection model, called HYFF-CB (Hybrid Feature Fusion Visual Model for Cargo Boxes). This model combines key technologies, such as a positioning attention mechanism, a fusion-enhanced pyramid structure, and a synergistic weighted loss system, so as to give full play to the advantages of YOLOv8 in object detection, while making up for its deficiencies in special application scenarios.

## 2. Research Status

### 2.1. Automatic Loading and Unloading Technology

In the digital transformation process of the manufacturing and logistics industries, automatic loading and unloading technology is the key to achieving full-process automation. However, this technology is currently stagnated at the early stage of development, due to technical challenges, the diversity of market demands, and insufficient logistics standardization. Against the backdrop of negative population growth in a large number of countries, manual loading and unloading is labor-intensive, enterprises face recruitment difficulties and rising labor costs, and logistics services have a significant impact on online shopping satisfaction [24,25,26]. Therefore, automatic loading and unloading technology has become a point of that weakness in the industry that urgently needs to be overcome. In addition, it has witnessed a surging market demand and has broad application prospects. Its potential market size has reached hundreds of billions to trillions [27,28].

A variety of automation solutions have emerged in the logistics field [29,30]. Among different types of cargo packaging, boxed goods account for about 46% of the automatic loading system market, with the largest demand [29,30]. However, problems such as the diversity of truck models, inconsistent sizes of unloading bins, and irregular stacking inside the truck cause high technical difficulties, which are the focus of this study.

In automatic loading and unloading technology, the truck scanning system is crucial. This system obtains the localization and spatial parameters inside trucks through technologies such as machine vision or LiDAR (Laser Radar), and transmits these parameters to the intelligent loading control system through specified communication protocols. Industrial three-dimensional (3D) cameras and LiDAR have gradually become the standard configurations of automatic loading and unloading systems. When loading and unloading goods of different shapes and sizes in a mixed manner, the existing systems fail to automatically adjust the loading and unloading strategy according to real-time conditions, which may result in low space utilization or inefficient loading and unloading.

### 2.2. CNN-Based Object Detection Algorithm

In recent years, object detection technology, driven by CNNs (convolutional neural networks), has shown excellent performance in image pattern recognition and classification tasks [31,32,33,34]. Object detection technology mainly uses two-stage and single-stage approaches, among which single-stage approaches are favored for their rapidity and efficiency. M Ju et al. constructed a CNN-based object detector, ISTDet (Infrared Small Target DETection), for infrared small-object detection [35].

Among single-stage object detectors, the SSD (Single Shot MultiBox) detector utilizes CNN’s multi-scale feature maps and predefined bounding boxes to detect objects of different scales [36]. YOLO models have been applied in various fields, such as transportation, biometrics, medical care, and agriculture [37,38,39,40,41,42,43,44,45,46,47,48]. They are also widely used in the object detection of drones and robots [49,50]. JEON-SEONG et al. integrated the generative adversarial network (GAN) and YOLOv5 into an end-to-end structure, and used image enhancement to solve the problem of insufficient lighting inside freight trucks. Their experimental results showed that the image detection for boxes inside freight trucks achieved an accuracy of 91.3% and a recall rate of 82% under poor lighting conditions [51]. Xiang et al. applied YOLOv8 to the automatic docking system for LNG loading arms [52]. Although YOLO models have shown broad application potential in various fields, object detection technology is still in its infancy and lacks mature solutions for loading and unloading scenarios, particularly when dealing with tasks such as box detection in complex environments.

To address this challenge, this study proposes a vision algorithm called HYFF-CB. In order to achieve a reasonable trade-off between computing resources and costs, based on YOLOv8, this algorithm integrates key technologies, such as an LA (localization attention) mechanism, a FEP (fusion-enhanced pyramid) structure, and a SW-Loss (synergistic weighted loss) system. It aims to improve the accuracy and robustness of detection of boxes and other cargo inside trucks. The HYFF-CB algorithm can effectively deal with actual loading and unloading scenarios, such as the diversity of truck models, inconsistent sizes of unloading bins, irregular stacking inside the truck, and complex lighting and weather conditions, thereby meeting the real-time and accuracy requirements of automatic loading and unloading systems for cargo detection. Through these technological innovations, the HYFF-CB algorithm is expected to promote the application of object detection technology in the loading and unloading field and provide new solutions for logistics automation.

## 3. Methods

**Figure 1:**
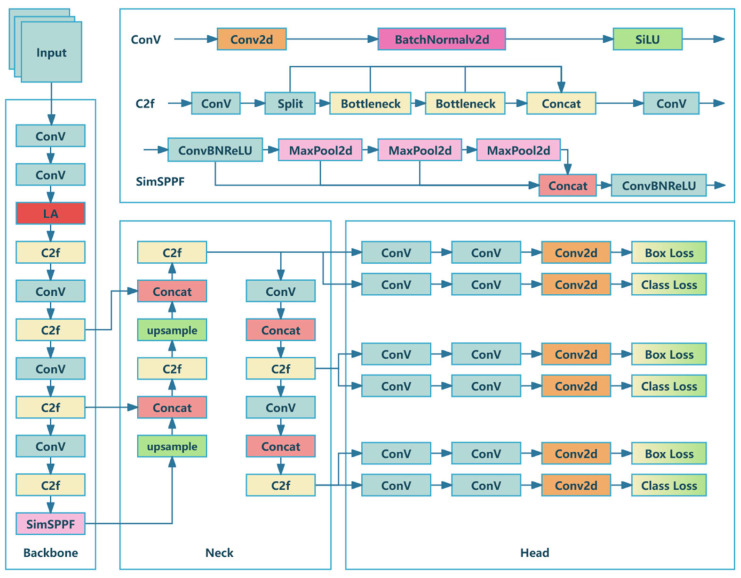
Framework of HYFF-CB model.

By investigating the images of boxes in loading and unloading scenarios, we found various problems concerning the boxes inside trucks, such as relatively small and non-uniform sizes, large quantities, severe occlusion with each other, irregular stacking inside the truck, and many types of SKUs (stock-keeping units). These problems result in poor detection performance and low accuracy of the model. Therefore, to balance the real-time and accuracy requirements of cargo loading and unloading, based on YOLOv8, HYFF-CB incorporates LA into the backbone and adopts SimSPPF [53], with a more simplified structure and faster inference speed, to meet the real-time requirements of the cargo box detection task. In the head, an FEP structure is adopted to fuse the features of objects at different scales. Finally, a SW-Loss system is incorporated to adapt to complex practical scenarios. Through experiments and practical tests, the HYFF-CB can effectively solve the above-mentioned problems and achieve a balance between speed and accuracy. The framework structure of HYFF-CB is shown in Figure 1.

### 3.1. LA Mechanism

In actual box storage and transportation scenarios, boxes are stacked in complex and diverse ways. They may have different angles, layers, and arrangements. Existing channel attention mechanisms, such as the SE (Squeeze-and-Excitation) module, can improve model performance to some extent. However, they often ignore important location information required to generate spatially selective attention maps while compressing global spatial information. Although spatial attention mechanisms (e.g., GeNet and GcNet) focus on spatial information, their ability to capture global information is relatively weak.

In box detection, however, location information and global information are equally crucial. When boxes are stacked densely, it is impossible to accurately distinguish the boundary locations of each box based solely on location information. If spatial location features are not fully considered, the box locations may be inaccurately detected, or multiple boxes may be misidentified as one. In addition, box detection based solely on local spatial information may cause the model to ignore distant or partially occluded boxes, thereby affecting the completeness and accuracy of detection.

**Figure 2:**
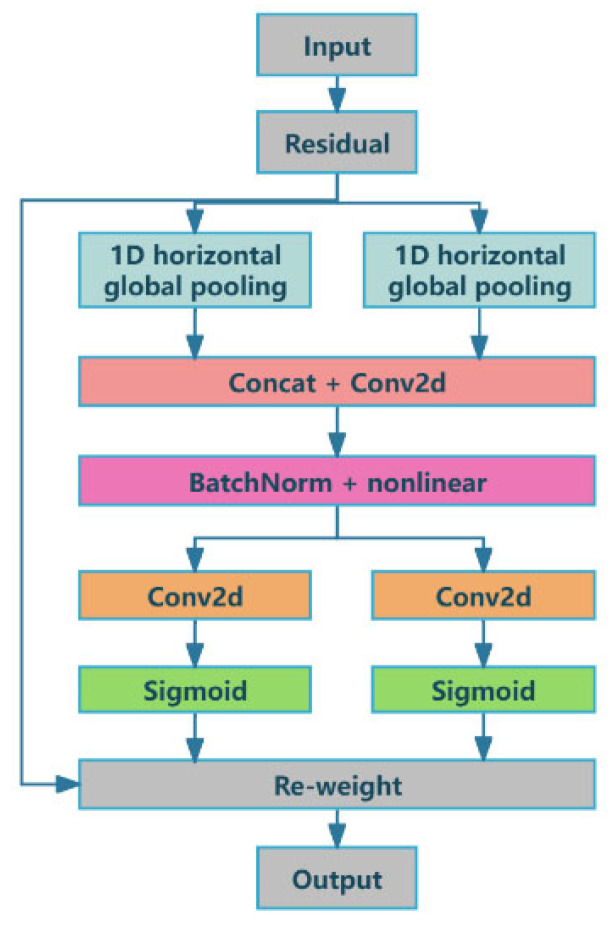
Structure of LA mechanism.

Since existing attention mechanisms cannot deal with the overall planning of location information and global information, the HYFF-CB model introduces an LA mechanism. As shown in Figure 2, the LA mechanism divides the attention process into two one-dimensional (1D) feature encoding processes to collect features along dual spatial dimensions. By capturing spatial information, the LA mechanism improves the precise localization of the regions of interest (boxes) and enables a better understanding of the spatial relationship between different regions in the input image. This allows the model to better distinguish the individual characteristics of each box in the case of complex box stacking, instead of misidentifying a pile of boxes as a whole or missing some of them.

With an innovative dual 1D feature encoding strategy, the LA mechanism significantly improves the performance of cargo detection and effectively overcomes the shortcomings of traditional attention mechanisms. By independently encoding features along two spatial dimensions, the LA mechanism is able to construct an attention map with precise localization awareness, thereby enhancing the model’s ability to represent the objects without increasing any computational burden. During the feature encoding process, the resulting feature map is encoded into an attention map with direction awareness and location awareness, thereby enhancing the ability to represent the objects of interest (boxes) and cleverly avoiding a large amount of computational overhead. The receptive field of the model is increased as well.

This paper assumes that the original feature map is $X\in R^{H\times W\times C}$, where H denotes the height, W denotes the width, and C denotes the number of channels. It is encoded along horizontal and vertical dimensions.

First, feature aggregation is performed on each channel c in the horizontal direction. The encoding function in the horizontal direction is set as $E_{h}$, and then the horizontal feature $Z^{h}\in R^{H\times 1\times C}$ is obtained. Similarly, feature aggregation is performed on each channel c in the vertical direction, to obtain the vertical feature $Z^{w}\in R^{1\times W\times C}$. The outputs of the pooling in two spatial dimensions are concatenated and go through a 1×1 convolutional layer, as shown in Equation (1):

(1) $$f=\delta \left(F1\left(\left[z^{h},z^{w}\right]\right)\right),\;f\in R^{C/r\times \left(H+W\right)}$$

where z denotes the concatenation operation along a spatial dimension; $\delta \left(\cdot \right)$ is a nonlinear activation function; and r is the reduction rate that controls the block size.

Then, f is decomposed into independent tensors. As shown in Equations (2) and (3), two other 1×1 convolution transforms, i.e., $F_{h}$ and $F_{w}$, are used to transform $f^{h}\in \;R^{C/r\times H}$ and $f^{w}\in \;R^{C/r\times W}$, respectively, into the tensors that are consistent with the number of channels in the input X.

(2) $$g^{h}=\delta \left(F_{h}\left(f^{h}\right)\right)$$

(3) $$g^{w}=\delta \left(F_{w}\left(f^{w}\right)\right)$$

The number of channels is usually decreased using an appropriate reduction rate $r\left(r=4,8,16,32,\ldots \right)$ to reduce the complexity of the model overhead. After a 1×1 convolution, the attention weight data are calculated using $sigmoid\delta \left(-\right)$.

Finally, the input features are multiplied by the weights to generate an LA map that highlights the most informative regions in the input. This attention mechanism effectively captures the spatial relationships between different regions of the input, allowing the network to focus on the most relevant features. Then, the outputs of Equation (4), i.e., $g^{h}$ and $g^{w}$, are expanded and used as attention weights.

(4) $$y_{c}\left(i,j\right)=x_{c}\left(i,j\right)\times g_{c}^{h}\left(i\right)\times g_{c}^{w}\left(j\right)$$

At the feature encoding stage, the LA mechanism is designed with a process for encoding feature maps, to generate attention maps that are aware of directions and locations. This process not only enhances the representation of target objects, but also effectively avoids the loss of location information caused by global pooling operations. By decomposing the channel attention into two parallel 1D features, the LA mechanism successfully integrates spatial localization information into the generated channel attention feature vector.

In vision tasks, the capturing of the spatial structure requires preserving the location information of features. Convolution kernels with sizes $\left(H,1\right)$ and $\left(1,W\right)$ are obtained along the horizontal and vertical directions of the input feature map, respectively. These two convolution kernels can be understood as two-dimensional arrays, where H and W represent the sizes of the kernels along the horizontal and vertical directions, respectively. During the convolution operation, the kernels slide along the two directions of the input feature map and extract features along the two axes to generate the output feature map. Specifically, in the case of input x, the two spatial ranges of the pooling kernels $\left(H,1\right)$ and $\left(1,W\right)$ are used to encode the horizontal localization and vertical localization of each channel, respectively. The output of channel c can be expressed by Equations (5) and (6):

(5) $$z_{c}^{h}\left(h\right)=\frac{1}{W}\sum_{0\leq i\leq W}x_{c}\left(h,i\right)$$

(6) $$z_{c}^{w}\left(w\right)=\frac{1}{H}\sum_{0\leq i\leq H}x_{c}\left(j,w\right)$$

where $z_{c}^{h}\left(h\right)$ and $z_{c}^{w}\left(w\right)$ represent the output of channel *c* at height *h* and at width *w*, respectively. This function mapping aggregates features in two spatial directions to form a pair of direction-aware feature mappings. This method improves the precise localization of regions of interest by capturing spatial information. By incorporating such direction-aware feature mappings, the network can better understand the spatial relationships between different regions of the input, thereby improving the performance on vision tasks.

This series of technological innovations enables the LA mechanism to significantly improve the model’s ability to pay attention to spatial features, thus accurately improving object localization accuracy and more accurately capturing spatial dependencies. Ultimately, these improvements enable the LA mechanism to achieve excellent box detection performance in complex real-world scenarios.

### 3.2. FEP

In actual scenarios, the box size may vary. For example, there are both large container boxes and small turnover boxes in a warehouse scene. The fused feature map allows the model to accurately detect the boxes at different scales, reducing missed detection or false detection caused by size differences. Therefore, the HYFF-CB model uses an FEP structure at its bottleneck layer, which enables the network to better integrate the features of objects of different scales, thereby improving the detection performance of boxes of different scales.

This paper assumes the low-level feature map to be L and the size to be $h_{l}\times w_{l}\times c_{l}$ (where $h_{l}$ represents the height, $w_{l}$ represents the width, and $c_{l}$ represents the number of channels). After an upsampling operation, the FEP obtains $L_{up}$, and the size becomes $h_{up}\times w_{up}\times c_{up}$, as shown in Equation (7):

(7) $$L_{up}\left(i,j,k\right)=\sum_{m,n}\sum_{l}L\left(m,n,l\right)\times K\left(i-\alpha m,j-\alpha n,k-l\right)$$

where $\left(i,j,k\right)$ represents the location of the feature map after upsampling, $\left(m,n,l\right)$ represents the localization of the original feature map, K is the interpolation kernel function, and α is the upsampling factor.

This paper assumes that the high-level feature map is H and the size is $h_{h}\times w_{h}\times c_{h}$. After a downsampling operation, $H_{down}$ is obtained, and the size becomes $h_{down}\times w_{down}\times c_{down}$, as shown in Equation (8):

(8) $$H_{down}\left(i,j,k\right)=\underset{m,n}{\max}\sum_{l}H\left(m,n,l\right)$$

where $\left(i,j,k\right)$ represents the localization of the feature map after downsampling, $\left(m,n\right)$ is the localization within the pooling window, and l represents the channel dimension.

The upsampled low-level feature map $L_{up}$ and the downsampled high-level feature map $H_{down}$ are fused into the feature map F, as shown in Equation (9):

(9) $$F\left(i,j,k\right)=L_{up}\left(i,j,k\right)+H_{down}\left(i,j,k\right)$$

Since the fused features contain rich semantic and localization information, the model can more accurately determine the locations and categories of boxes during detection. This fusion method also enhances the robustness of the model, allowing it to better cope with various complex scene changes, such as lighting changes and partial occlusion of boxes. For example, even if a box is partially occluded by other objects, the model can still use the fused features to infer the category of the occluded part based on semantic information, and determine the boundaries of the unoccluded part based on localization information, thereby achieving higher accuracy in box detection.

### 3.3. SW-Loss System

During the loss calculation process, an SW-Loss system is created to address various problems in box detection, and is used to replace the original loss function, aiming to effectively deal with the main contradictions in box detection.

In complex scenes, such as dense box stacking, partial box occlusion, and lighting changes, it is difficult to accurately detect boxes by relying solely on classification or regression. Classification can better identify boxes based on the location and shape information provided by regression, whereas regression can provide more accurate localization and size estimation based on the target objects identified by classification. This mutually complementary relationship enables the model to more comprehensively understand the box information in the image, thereby completing the box detection task more accurately.

Therefore, the SW-Loss system consists of two parts: classification and regression. The classification part uses BCE (binary cross-entropy), and the regression part uses DFL (distribution focal loss) [36] and the CloU loss function, as shown in Equation (10):

(10) $$L_{SW}=\lambda_{cls}\cdot L_{BCE}+\lambda_{dfl}\cdot L_{DFL}+\lambda_{ciou}\cdot L_{CIoU}$$

where $\lambda_{cls}$, $\lambda_{dfl}$, and $\lambda_{ciou}$ are the weight coefficients of BCE loss, DFL loss, and CloU loss, respectively.

BCE is used for classification tasks, as shown in Equation (11):

(11) $$BCE=-\frac{1}{N}\sum_{i=1}^{N}\left[y_{i}\cdot \log\left(p\left(y_{i}\right)\right)+\left(1-y_{i}\right)\cdot \log\left(1-p\left(y_{i}y_{i}\right)\right)\right]$$

where $y_{i}$ is the true label, $p\left(y_{i}\right)$ is the probability of the positive class in model prediction, and N is the total number of samples. In box detection, BCE helps the model to distinguish the boxes from the background. In particular, when the contrast between the box and the background is not high, or the box is partially blocked, BCE improves the model’s ability to identify boxes by focusing on the classification accuracy of each pixel.

DFL is used for regressing the bounding boxes. Its core idea is to model the location of a bounding box as a probability distribution, rather than a fixed value. DFL is given by Equation (12):

(12) $$DFL=-\sum_{i=0}^{n}\left[\left(y_{i+1}-y\right)\log\left(S_{i}\right)+\left(y-y_{i}\right)\log\left(S_{i+1}\right)\right]$$

where $y_{i}$ and $y_{i+1}$ are the discretized locations of the bounding box, and $S_{i}$ and $S_{i+1}$ are the probabilities of the corresponding locations. DFL is designed for difficult-to-classify samples in object detection. Its core idea is to reduce the weights of easy-to-classify samples and increase the weights of difficult-to-classify samples. It encourages the model to pay more attention to those difficult-to-detect boxes (e.g., boxes that are similar to the background, have blurred boundaries, or are partially occluded), thereby improving the model’s detection accuracy for these boxes.

CloU considers the penalty terms for the inconsistency between the prediction box and ground truth box in intersection over union (IoU), distance between centroids, and aspect ratio, as shown in Equation (13):

(13) $$CIoU=1-IoU+\frac{d^{2}}{c^{2}}+\alpha v$$

where d is the distance between the centroids of the prediction box and ground truth box, c is the diagonal length of the minimum bounding rectangle, v is a correction factor used to consider the difference in aspect ratio between the prediction box and ground truth box, and α is a balance factor. In box detection, the shapes and sizes of boxes may vary depending on the cargo loaded. The CloU loss function uses these additional penalty terms to enable the model to more accurately predict the locations and sizes of boxes.

## 4. Experimental Results

### 4.1. Dataset

We collected a high-resolution image dataset specifically for box detection. The dataset consists of 9534 images; the dataset was randomly split into a training set (7627 images), a validation set (953 images), and a test set (954 images). The images were taken at Milaotou Warehouse and Vinda Paper Warehouse. Each image had 10 to 100 target boxes.

Data collection covered a variety of onsite application scenarios, including the following:

1. Images from various shooting angles, including images of boxes with varying degrees of blur and occlusion;
2. Various lighting conditions, including natural light, artificial light, forward light, and backlight;
3. Various weather conditions, such as sunny, cloudy, and rainy days;
4. Various locations, including warehouses, trucks, and outdoor scenes.

The statistical results of the dataset’s quantities according to shooting locations are shown in Table 1.

The dataset from the warehouse is the largest. The illumination in the warehouse is relatively uniform, resulting in the lowest detection difficulty. The illumination in the dataset of trucks is also relatively even, yet there is frequent occurrence of occlusion by porters. The outdoor dataset is relatively small, and pictures with extreme lighting conditions, such as backlighting and low illuminance, are likely to appear, which makes the detection more difficult.

**Figure 3:**
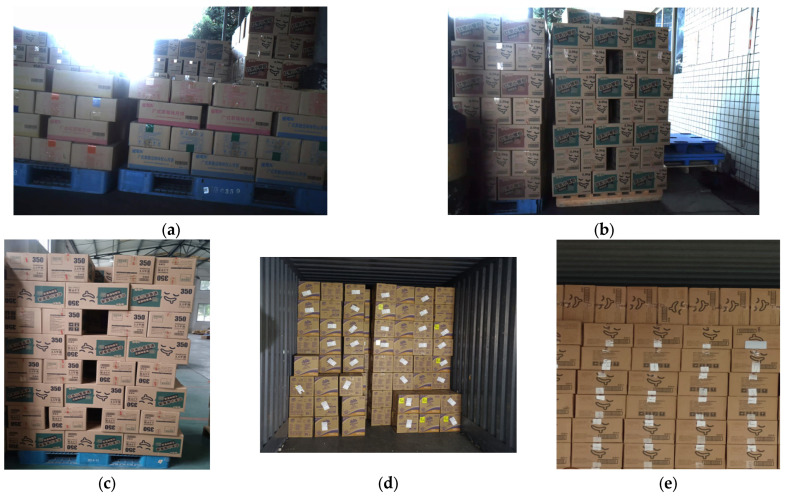
Images of boxes in different environments. (**a**) Backlight environment. (**b**) Outdoor environment. (**c**) Warehouse environment. (**d**) Truck interior under fill light. (**e**) Truck interior under natural light.

We ensured that the dataset could cover various practical application scenarios of boxes. Changes in these conditions brought greater challenges to box detection in images, as shown in Figure 3.

**Figure 4:**
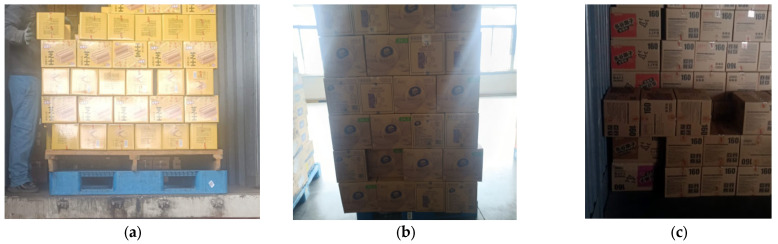
Cargo box pictures under extreme lighting conditions. (**a**) Light reflection interference. (**b**) High-contrast background. (**c**) Low illuminance.

The boxes involved in the dataset could face challenges such as stacking, rotation, and tilt. Other objects or shadows in the images could cause the boxes to be partially obscured. To enhance the generalization ability of the model, we included the images under more challenging scenarios, such as reflected light interference, high-contrast backgrounds, and extreme lighting conditions, into the dataset. Each of these factors made it more difficult to identify the box edges, as shown in Figure 4. The in-depth analysis and simulation results for these complex scenarios show that the object detection model can yield higher detection accuracy and robustness amid diversity and uncertainty.

**Figure 5:**
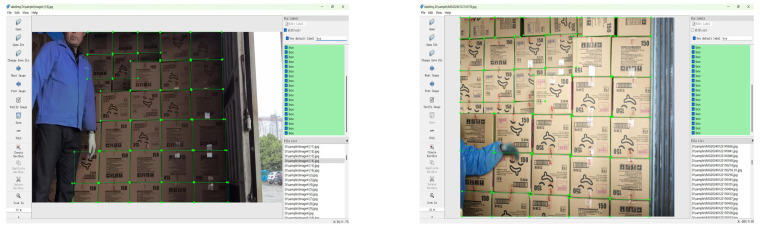
Labeled images.

All datasets were manually labeled using LabelImg. The labeled images are shown in Figure 5.

### 4.2. Experimental Process and Evaluation Indicators

This dataset contains 9534 images, and the data samples are divided in a ratio of 8:1:1. To determine the optimal hyperparameter configuration, a sensitivity analysis method was adopted, and the model performance was evaluated by changing the basic learning rate, optimizer, weight decay, and other parameters one by one. The equipment and some of the hyperparameters used in the experiments are listed in Table 2.

The SW-Loss system includes two parts, namely, classification and regression. To balance these two factors, we first set the classification score of a candidate sample to be $S_{cls}$, the regression score to be $S_{reg}$, and the weights to be $w_{cls}$ and $w_{reg}$ for SW-Loss in the selection of samples. Then, the comprehensive score was $S=w_{cls}S_{cls}+w_{reg}S_{reg}$. After calculating the comprehensive score S for all candidate samples, we selected the top N samples with higher comprehensive scores as positive samples P, and regarded the remaining samples as negative samples (i.e., the negative sample set N).

Usually, the AP (average precision), AR (average recall), and mAP (mean average precision) are used to evaluate the matching degree of the model to the dataset. They are given by Equation (14):

(14) $$AP=\frac{TP}{TP+FP},\;AR=\frac{TP}{TP+FN},\;mAP=\frac{1}{N}\sum_{i=1}^{N}{AP}_{i}$$

where TP denotes the correctly recognized target, FP denotes the object that is not the target but is recognized as the target, and FN denotes the unrecognized target. Precision is the proportion of samples that are actually positive among the samples that are predicted to be positive. The recall rate is the proportion of samples that are correctly predicted to be positive among all positive samples. In this study, the IoU value between the prediction box and the ground truth box was greater than 0.5, implying that the predicted object was a real target. A higher recall rate indicates that the model can better identify real targets and miss fewer targets.

### 4.3. Ablation Experiment

The ablation experiment applied the principle of control variables to control a part of the network in the model, to help provide a better understanding of the network function. Therefore, in this study, each module was added one by one to verify the impact of the improved modules on the model. The experimental results are shown in Table 3. Two modules were added one by one to the original model and compared with the original model. When adding attention modules, the commonly used attention modules SE (Squeeze-and-Excitation) and CBAM (Convolutional Block Attention Module) were selected for comparison with LA to verify the impact of the improved module on the model performance.

As observed from Table 3, the introduction of each individual improvement led to a certain degree of performance enhancement.

The attention modules were added to the YOLOv8s basic model. Three different attention mechanisms, SE, CBAM, and LA, were added. After adding LA, the performance of the model improved the most. The relative improvements in AP, AR, and mAP@50% were 0.74%, 0.74%, and 0.31%, respectively. This indicates that LA can enhance the model’s ability to focus on spatial features. Compared with other attention modules, LA has a stronger ability to detect boxes.

After adding the FEP module, the relative improvements in AP and AR were 0.53% and 0.63%, respectively, which shows that the FEP module can improve the model’s detection performance for boxes of different scales. After adding the SW-Loss module, the relative improvements in AP and AR were 0.21% and 0.21%, respectively. After adding the FEP and SW-Loss modules, compared with the basic model, the relative improvements in AP, AR, and mAP were 0.63%, 0.74%, and 0.2%, respectively.

After adding the FEP, SW-Loss, and LA modules, compared with the basic model, the relative improvements in AP, AR, and mAP were 1.37%, 1.37%, and 0.51%, respectively. The floating point operations per second (FLOPs) only increased by 0.5 GFLOPs/s compared with the basic model, an increase of 1.7%, indicating little impact on the detection speed.

In conclusion, these improvement measures can comprehensively enhance the model’s learning and generalization abilities in complex loading and unloading scenarios.

### 4.4. Comparative Experiment

**Figure 6:**
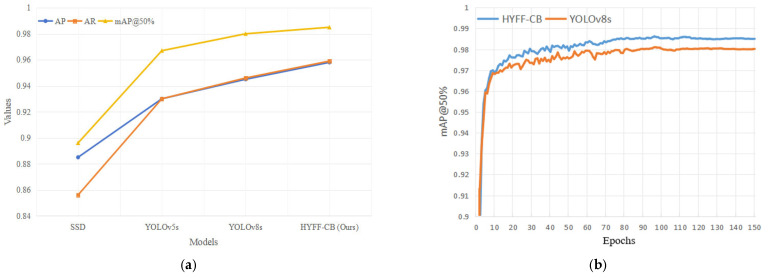
Results of model performance comparison. (**a**) Curve of model performance comparison. (**b**) mAP curve.

We selected SSD, YOLOv5s, YOLOv8s, and HYFF-CB for model performance comparison. The same training parameters were used to verify the effectiveness of the model proposed in this paper. The results are shown in Table 4 and Figure 6. Figure 6a is a curve graph comparing the performance of each model, which contrasts AP, AR, and mAP@50%. Figure 6b displays the performance comparison between the YOLOv8s and HYFF-CB models after 150 epochs. The abscissa represents the number of training rounds, the ordinate represents mAP@50%, and the display range is limited to 0.9–1.

It can be seen from the table above that the mAP of our HYFF-CB model was 0.5% higher than the original YOLOv8s model under basically the same computing amount. Compared with the classic YOLOv5s model, the mAP of our model increased by 1.8%. Therefore, the improved model in this paper can meet the box detection needs of automatic loading and unloading systems.

### 4.5. Validation of Actual Effect

**Figure 7:**
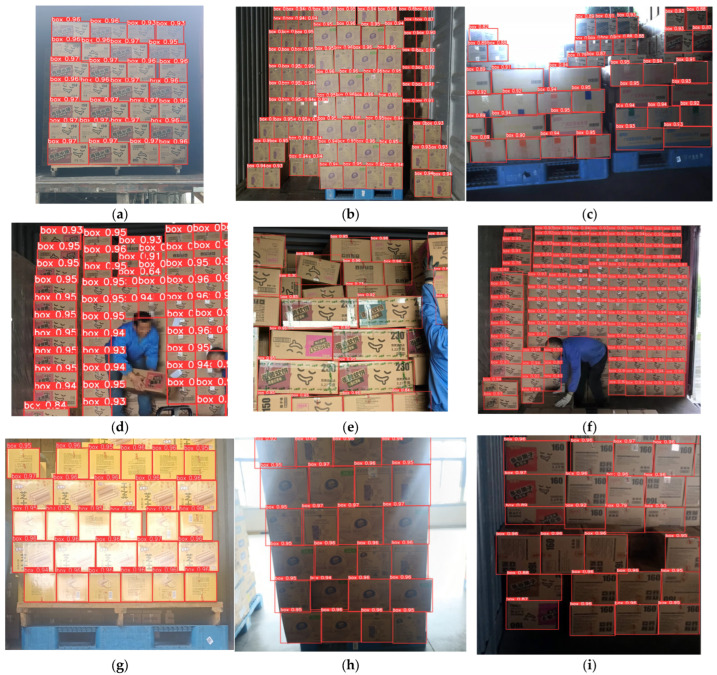
Actual detection effect diagram. (**a**) Truck + natural light. (**b**) Truck + supplementary light. (**c**) Backlight. (**d**) Occlusion. (**e**) Irregular stacking. (**f**) Small targets. (**g**) Light reflection interference. (**h**) High-contrast background. (**i**) Low illuminance.

To verify the actual effectiveness of the HYFF-CB model, images of various scenarios were collected using an industrial camera. Tests were carried out on these scenarios. A total of 663 test samples were selected, and the detection results of the HYFF-CB model were verified manually. The detection results of each cargo box were checked one by one, and the real precision rate and recall rate were counted. The results are shown in Table 5 and Figure 7.

Here, TP represents the number of correctly detected cargo boxes, FP represents the number of objects that are not cargo boxes but are detected as cargo boxes, FN represents the number of missed detected cargo boxes, RP (real precision) is the real precision rate, counted manually, and RR (real recall) is the real recall rate, counted manually. The real precision rate reached 99.9%, and the real recall rate reached 99.8%, indicating that the precision rate and recall rate of the HYFF-CB model can already adapt to various scenarios in practical applications.

The test scenarios included natural light in trucks, supplementary lighting in trucks, backlit scenarios, small-sized cargo box targets, occlusion, irregular stacking, light reflection interference, low illuminance, etc. The test results show that the model can accurately predict the location of each box, and always maintains high reliability.

## 5. Conclusions

In the process of the automation development of the manufacturing and logistics industries, the accurate and efficient implementation of automatic loading and unloading technology is of great significance. This paper focuses on this issue. By using an industrial camera to collect carriage images in real time, and innovatively integrating the LA attention module into the classic YOLOv8 model, accurate detection of cargo boxes in the carriage was carried out. This model could not only clearly identify the stacking positions of the cargo boxes, but also accurately count their quantities.

After a large number of rigorous tests and in-depth comparative analysis of the detection effects of various different models, it was found that the HYFF-CB model proposed in this paper exhibits excellent performance, and its detection rate is significantly higher than that of other models. The detection performance and practical effects of this model meet or even exceed the expected requirements, and the model is fully capable of perfectly adapting to various complex application scenarios involved in automatic loading and unloading, strongly promoting the progress of automatic loading and unloading technology from theory to practical application.

Although the HYFF-CB model proposed in this paper successfully meets the basic requirements of practical applications in terms of detection accuracy and recall rate, in order to further enhance the practicality and commercial value of this technology in the field of automatic loading and unloading, especially to better match the refined operations of robotic arms, we have clearly planned the next research direction. In the follow-up, we will focus on deeply integrating and matching the HYFF-CB model with the depth information of the cargo boxes scanned by LiDAR. By accurately extracting and efficiently transmitting the spatial position information of the cargo boxes to the robotic arm, a high degree of automation and intelligence of the entire loading and unloading process can be achieved, thus accelerating the achievement of commercial standards for automatic loading and unloading, and injecting new vitality into the development of the industry.

## Figures and Tables

**Table 1:** Dataset quantity at each shooting location.

Location	Warehouse	Truck	Outdoor	Total
**Quantity**	5962	2281	1291	9534
**Proportion**	62.5%	23.9%	13.6%	100%

**Table 2:** The equipment and some of the hyperparameters used during the experiments.

Device	Parameter	Hyperparameters	Value
CPU	Intel(R) Core(TM) I7-12650H	Optimizer	SGD
RAM	32 GB	Epochs	150
GPU	NVIDIA GeForce RTX 4060	Batch size	16
GPU memory	8 GB	Imgsz	640
Operating System	Windows 11	Workers	8
Deep learning framework	PyTorch 2.1.2	lrf	0.01
Programming language	Python 3.8.18	weight_decay	0.0005

**Table 3:** Ablation experiment.

FEP	SW-Loss	LA	CBAM	SE	AP (%)	AR (%)	mAP@ 50%	FLOP GFLOPs/s	Parameters (M)	Model Size (M)
一	一	一	一	一	94.5	94.6	98.0	28.6	11.1	21.4
一	一	一	一	√	94.5	94.8	98.0	28.7	11.2	21.5
一	一	一	√	一	94.6	95.0	98.1	28.9	11.3	21.9
一	一	√	一	一	95.2	95.3	98.3	28.7	11.2	21.7
√	一	一	一	一	95.0	95.2	98.1	28.9	11.4	21.9
一	√	一	一	一	94.7	94.8	98.1	28.6	11.1	21.4
√	√	一	一	一	95.1	95.3	98.2	29.0	11.4	22.0
**√**	**√**	**√**	**一**	**一**	**95.8**	**95.9**	**98.5**	**29.1**	**11.6**	**22.3**

“√ ” indicates that this module was introduced; “一” indicates that this module was not introduced.

**Table 4:** Performance comparison experiment for different detection models.

Model	Epochs	AP (%)	AR (%)	mAP@50%	FLOPs GFLOPs/s
SSD [37]	150	88.5	85.6	89.6	22.0
YOLOv5_G [51]	150	90.7	80.4	87.8	24.0
YOLOv5s	150	93.0	93.0	96.7	24.2
YOLOv8s	150	94.5	94.6	98.0	28.6
**HYFF-CB (ours)**	**150**	**95.8**	**95.9**	**98.5**	**2** **9.1**

**Table 5:** Manual verification of model’s detection effect.

Number of Test Samples	TP	FP	FN	RP	RR
663	15,264	16	25	99.9%	99.8%

## Data Availability

The data presented in this study are available on request from the authors; the data underlying the results presented in this paper are not publicly available at this time.
